# PitSurgRT: real-time localization of critical anatomical structures in endoscopic pituitary surgery

**DOI:** 10.1007/s11548-024-03094-2

**Published:** 2024-03-25

**Authors:** Zhehua Mao, Adrito Das, Mobarakol Islam, Danyal Z. Khan, Simon C. Williams, John G. Hanrahan, Anouk Borg, Neil L. Dorward, Matthew J. Clarkson, Danail Stoyanov, Hani J. Marcus, Sophia Bano

**Affiliations:** 1 Department of Computer Science, University College London, London, UK; 2 Wellcome/EPSRC Centre for Interventional and Surgical Sciences, University College London, London, UK; 3 Department of Medical Physics and Biomedical Engineering, University College London, London, UK; 4 Department of Neurosurgery, National Hospital for Neurology and Neurosurgery, London, UK

**Keywords:** Surgical scene understanding, Segmentation, Landmark detection, Pituitary tumour

## Abstract

**Purpose:**

Endoscopic pituitary surgery entails navigating through the nasal cavity and sphenoid sinus to access the sella using an endoscope. This procedure is intricate due to the proximity of crucial anatomical structures (e.g. carotid arteries and optic nerves) to pituitary tumours, and any unintended damage can lead to severe complications including blindness and death. Intraoperative guidance during this surgery could support improved localization of the critical structures leading to reducing the risk of complications.

**Methods:**

A deep learning network PitSurgRT is proposed for real-time localization of critical structures in endoscopic pituitary surgery. The network uses high-resolution net (HRNet) as a backbone with a multi-head for jointly localizing critical anatomical structures while segmenting larger structures simultaneously. Moreover, the trained model is optimized and accelerated by using TensorRT. Finally, the model predictions are shown to neurosurgeons, to test their guidance capabilities.

**Results:**

Compared with the state-of-the-art method, our model significantly reduces the mean error in landmark detection of the critical structures from 138.76 to 54.40 pixels in a 1280 $\times$ 720-pixel image. Furthermore, the semantic segmentation of the most critical structure, sella, is improved by 4.39% IoU. The inference speed of the accelerated model achieves 298 frames per second with floating-point-16 precision. In the study of 15 neurosurgeons, 88.67% of predictions are considered accurate enough for real-time guidance.

**Conclusion:**

The results from the quantitative evaluation, real-time acceleration, and neurosurgeon study demonstrate the proposed method is highly promising in providing real-time intraoperative guidance of the critical anatomical structures in endoscopic pituitary surgery.

## Introduction

**Fig. 1:**
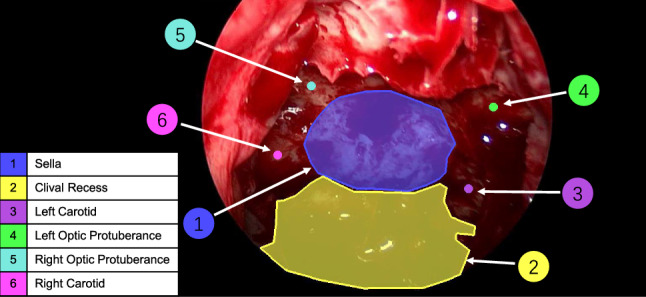
Critical anatomical structures as present during the sellar phase of endoscopic pituitary surgery. Sella (1) and Clival Recess (2) are relatively bigger structures and easy to identify. Carotids (3 left, 6 right) and Optic Protuberance (4 left, 5 right) are critical neurovascular structures to be avoided

The pituitary gland secretes hormones essential for supporting human life. It is a pea-sized gland found at the base of the brain close to critical nerves and vessels [1, 2]. Tumours formed on this gland may lead to symptoms such as vision loss and changes in bodily function [3]. Endoscopic pituitary surgery is the gold-standard method for removing pituitary tumours [3]. With the assistance of an endoscope to visualize the procedure, surgeons enter the sella through the nasal cavity, with the aim of maximizing tumour resection whilst minimizing collateral damage to critical surrounding anatomical structures [3]. Identifying these critical structures is challenging, as the structures are located behind the sphenoid bone so their position can only be inferred from the their imprints on the bone (see Fig. 1) [1, 2]. Failure to distinguish the safe sella region, behind which the tumour is located, from the other structures may lead to adverse effects [2].

During the surgery, the most common way to locate these anatomical landmarks involves using an optical tracking system [4]. This system registers the patient’s head to a 3D magnetic resonance image before the operation, and then a tracked pointer is used to identify the specific areas of the sella and landmarks of other critical structures on the sphenoid bone [4]. However, if the surgeon’s view is obstructed by blood or if they remove and then replace the endoscope in the sphenoid sinus, they may need to re-identify these critical anatomical structures. Moreover, this process is time-consuming, disrupts surgical workflow, and can also bring in potential risks [2, 5].

Deep learning encoder–decoder architectures find extensive application in tasks such as image segmentation and landmark detection [6]. Convolution neural networks (CNNs) are often used in the encoder phase to extract features from images, progressively reducing the resolution of the feature maps. Subsequently, the decoding phase involves gradual image reconstruction through upsampling or deconvolution. In [7], a pyramid scene parsing network (PSPNet) was trained to localize the safe and dangerous areas of dissection and anatomical landmarks during the laparoscopic cholecystectomy. In [8], an UNet model was trained to predict the positions of critical anatomical structures in the nasal phase of endoscopic pituitary surgery. In [9], a multi-task network (PAINet) was proposed to identify the areas of the sella and clival recess, and central points of eight other critical structures during the sellar phase of endoscopic pituitary surgery. Based on a U-Net++ [10] architecture with an EfficientNetB3 encoder [11], PAINet achieved state-of-the-art performance on the dataset. However, while PAINet managed to give temporally consistent segmentation performance, the detection of landmarks required extensive improvement.

Moreover, real-time inference is always needed for intraoperative guidance in clinical settings. In general, the pursuit of ever more accurate results frequently results in highly complex deep learning architectures that are computationally costly and slow [12]. One approach to this issue is to design more efficient architectures by using techniques such as downsampling and upsampling [13], efficient convolution [14], residual connection [15]. In [16], by holistically and efficiently aggregating spatiotemporal knowledge through the convolutional long short-term memory (LSTM) layers, the method achieves real-time instrument segmentation. In [17], authors designed an encoder–decoder architecture featuring a residual block with a squeeze-and-excitation network, achieving real-time segmentation in colonoscopy with fewer parameters. Despite achieving real-time performance with well-designed architectures, these methods face constraints, especially when utilizing common backbone networks in deep learning models, where backbone architectures usually remain unchanged [12]. In these cases, using model acceleration techniques [18] is a more flexible solution in practice, which can reduce the dependence on the network structure design. Among these methods, open neural network exchange (ONNX)^1^ and NVIDIA TensorRT^2^ are most widely used. ONNX is a standard for model interchange, allowing models to be shared between different deep-learning frameworks. TensorRT, on the other hand, is focused on accelerating the inference of deep learning models on NVIDIA GPUs.

Improving on the downfalls of PAINet [9], PitSurgRT is proposed. Our contributions are as follows:

1. Based on the HRNet [19] which originally solved the segmentation task only, we proposed a multi-task network named PitSurgRT that can solve the anatomy segmentation and landmark detection simultaneously in the endoscopic pituitary surgery.
2. An effective loss function that combines four losses is proposed in this paper to solve the issue of the highly imbalanced dataset for the training.
3. Through the fivefold cross-validation based on the in-vivo dataset, the proposed PitSurgRT is demonstrated to significantly improve the landmark detection and semantic segmentation of the critical anatomical structures in the sellar phase of endoscopic pituitary surgery compared with our previous work PAINet.
4. The proposed model is accelerated by using the TensorRT technique to achieve real-time intraoperative guidance. The results are verified by 15 neurosurgeons.

To the best of our knowledge, this is the first work that can achieve real-time and simultaneous inference of anatomy segmentation and landmark detection in endoscopic pituitary surgery.

## Methodology

### Clinical motivation and validation

The pituitary tumour is found behind the sella, highlighted in blue in Fig. 1, and is required to be opened during endoscopic pituitary surgery. The clival recess, highlighted in yellow in Fig. 1, is not as surgically important as the other structures. However, as it is a large, visually distinct, and common structure, it provides additional visual information to help locate the other structures while training the model.

Our problem is focused on landmark detection of four critical structures, namely the left and right carotid and the left and right optic protuberance, shown in Fig. 1. Since these structures are small with poorly defined boundaries, segmentation is not desired. The remaining 4-structures detected in [9] have not been used in this study as they are considered less important for the surgery compared to other structures.

The proposed model is measured by the performance of: (i) the semantic segmentation of the sella and clival recess, using the mean interval over union (mIoU) metric; and (ii) the landmark detection of the remaining 4-structures, using the mean percentage of correct keypoints within 20% (MPCK20) and mean distance (mDistance) metrics. MPCK20 is the percentage of predicted landmarks that fall within a 144-pixel (20% of the image) radius of the ground truth, mean-averaged across the 4 structures. mDistance is the Euclidean distance between the predicted and the ground-truth landmarks, mean-averaged across the 4 structures.

**Clinical Validation:** As many images in our dataset are not fully labelled, (i.e. some anatomical structures do not have ground truth on images), a clinical validation test was conducted on the proposed model validation outputs. 10 predicted outputs (without ground-truth labels) from 10 randomly selected patients were selected. For each landmark predicted on each image, the neurosurgeons were requested to rate them on a scale of 1 to 5, where (1) not accurate at all; (2) somewhat accurate but acceptable; (3) sufficiently accurate to be acceptable; (4) accurate; (5) very accurate. Fifteen neurosurgeons (4 consultants, 11 trainees) evaluated the outputs by questionnaire.

### Proposed PitSurgRT

While an encoder–decoder architecture like UNet [20] has been demonstrated to be effective for segmentation tasks in biomedical images, the resolution reduction in continuous downsampling causes the loss of spatial detail information, especially in the pooling layers, which is detrimental to position-sensitive vision problems such as landmark detention [9, 19]. Therefore, to improve the performance of PAINet [9], especially for landmark detection, we utilize HRNet as the base model to maintain high-resolution representations. Compared with UNet++ structure used in PAINet, which maintains multi-resolution representation to improve spatial precision by continuously fusing low-resolution expression with high-resolution expression, HRNet not only maintains several resolutions in a parallel manner but also repeatedly fuses high and low resolutions between stages to boost high-resolution and low-resolution representations [19], which are beneficial for both segmentation and landmark detection tasks.

**Fig. 2:**
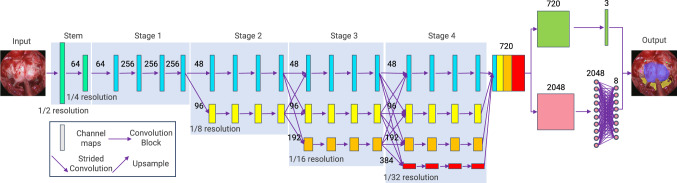
The architecture of PitSurgRT for simultaneous segmentation and landmark detection

The proposed PitSurgRT architecture is shown in Fig. 2. The input image first passes through a stem, and the image resolution is reduced to 1/4 of the original through two $3\times 3$ convolution operations with a step size of 2. Then, the output feature maps pass the main body of the HRNet consisting of four stages with four parallel convolution branches. The resolutions of feature maps in four branches are maintained as 1/4, 1/8, 1/16, and 1/32, respectively. The first stage of the HRNet consists of four residual units and the width of the output feature maps are 48 and 96 for the two branches formed in stage 2. Starting from the second stage, each branch will maintain a fixed feature map width. Every time from the previous stage to the next stage, the number of branches will increase by one, and the feature map width of the newly added branch is twice that of the previous branch. The final feature map widths of the four branches are 48, 96, 192, and 384, respectively. Between each two stages, the high-resolution feature maps and the low-resolution feature maps will be fused to exchange information. After the fourth stage, the low-resolution feature maps are upsampled and then fused with the output of the first branch to obtain a high-resolution feature map with 720 channels.

#### Multitask heads

Following the output feature map, two heads are connected that are responsible for the (a) segmentation of sella and clival recess and (b) landmark detection of the other four anatomies, respectively.

The segmentation head contains a $1\times 1$ convolutional layer with stride 1, followed by batch normalization and ReLU activation, and finally outputs the segmentation results by using a $1\times 1$ convolutional layer with stride 1, followed by argmax activation and upsampling to recover the original resolution for segmentation.

In terms of head for landmark detection, the output feature maps are passed through $1\times 1$ convolutional layer with stride 1, active average pooling with output size 1, ReLU activation, and fully connected layers to output the eight coordinates of four landmarks on the target image.

#### Combined losses for highly imbalanced dataset

While focal loss (FL) and mean squared error (MSE) loss are commonly used for imbalanced datasets and landmark detection in a variety of works, highly imbalanced class distributions are found in our datasets (see section “Data description and preparation” for details). Therefore, it is necessary to design a set of optimal combinations to achieve high-quality segmentation and landmark detection. Through extensive ablation studies (see section “Ablation study”), it was found the following combination of four losses is very effective for our tasks:

1 $$\begin{aligned} \textrm{Loss}=w_{\textrm{1}} \textrm{Dice}+w_{\textrm{2}} \textrm{BDL}+w_{3} \textrm{Wing}+ w_{4} \textrm{FL}, \end{aligned}$$

where the combination of Dice and boundary loss (BDL) are responsible for segmentation [21]. The combination of Wing loss [22] and focal loss (FL) is used for landmark detection. $w_{\textrm{1}}$, $w_{\textrm{2}}$, $w_{\textrm{3}}$, and $w_{\textrm{4}}$ are hyperparameters which are used to balance the losses.

The proposed method is implemented using PyTorch 1.12.0 on Python 3.8.17. The code is available here https://github.com/ZH-Mao/PitSurgRT.git.

### Model optimization for real-time guidance

While maintaining high-resolution representations and low representations together is promising to improve the performance of segmentation and landmark detection, such an architecture requires more computational resources and time, which is detrimental to intraoperative guidance that requires real-time performance.

Thanks to the model acceleration engine TensorRT, we can optimize and accelerate our proposed PitSurgRT on NVIDIA GPUs to achieve real-time performance. TensorRT offers options for accelerating trained models with various precisions such as 32-bit floating point precision (FP32), 16-bit floating point (FP16), and 8-bit integer precision (INT8). There is a trade-off between the model’s precision and efficiency. The open-source library torch2trt^3^ was used to convert our PyTorch model to the tensorRT engines directly.

## Experimental setup

### Data description and preparation

The dataset used in this study is the same as those used in [9], which consists of 635 frames obtained from 64 endoscopic pituitary surgery videos. Due to the challenges of annotating these images, the task was completed by four experts with consensus. Please refer to [9] for more details about annotation. Even so, there are still many anatomical structures that are not annotated. Except for the two largest areas sella and clival recess, only at most 65% of the data for the remaining anatomical structures have been annotated.

**Fig. 3:**
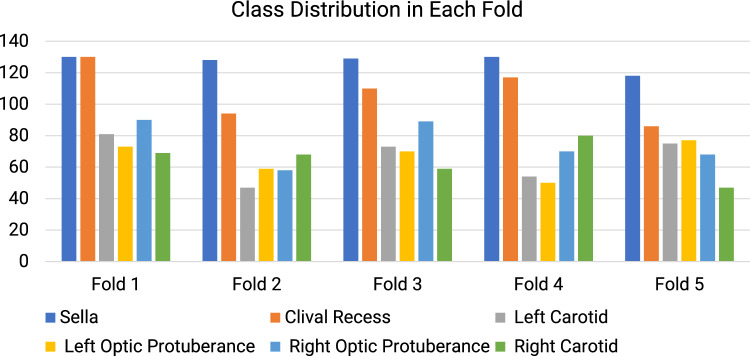
Class distribution per fold of the critical anatomical structures. The total number of images is 635

To validate the performance of the proposed model, fivefold cross-validation is used. Images are randomly split such that the number of structures in each fold is similar. Images from the same patients are put into the same fold, presenting in either the training or validation dataset. The class distribution over five folds is presented in Fig. 3.

### Training details

The training and validation processes are completed on an NVIDIA DGX A100 server on a single GPU with 48 GB memory. The proposed network is optimized by using an SGD optimizer during the training, with a momentum value of 0.9. The initial learning rate is set as 0.01 and a linearly decreasing scheduler is used for adjusting the learning rate every epoch. The minimum learning rate is 0.0001, and the model is trained for 500 epochs. For the first 300 epochs, only the segmentation head was trained while the landmark detection head was frozen. In the subsequent 200 epochs, both were trained simultaneously.

Given a limited number of annotated images for training, data augmentation is used. We shift images in the training dataset by up to 10% in any direction, zoom out or in images by up to 30%, and rotate them around the centre of images up to 30 degrees. In addition, we randomly increase or decrease brightness, contrast, and saturation by up to 30%, and hue by up to 10%. 50% of training data are augmented in each epoch. In addition, since many images in our datasets are not fully labelled, we use a sampler in our data loader to increase the probability of images with more anatomical structures on them. The probability is equal to the reciprocal of the number of images with the same number of anatomical structures.

### Ablation study

To find the best combination of losses and their corresponding weights, extensive ablation studies are conducted. We start with the same loss combination as that used in PAINet, i.e. (FL + MSE), followed by testing various loss combination strategies, which are listed in the first column in Table 2. For the preliminary tests about loss combination, the weight parameters are added empirically so that all losses are of the same order of magnitude. After the best combination of losses is found, the weight parameters are fine-tuned.

In addition, we also investigate the precision selection during the model optimization process using tensorRT. We compare the results of the original model, the converted model with FP32 precision, and the converted model with FP16 precision in terms of both accuracy and inference speed.

## Results and discussion

**Table 1:** Comparison with PAINet (fivefold cross-validation, results are displayed as $\mathrm{mean\pm std}$)

Model	Loss	Sella	Clival recess	Sella	Clival recess	Landmarks	
		IoU(%)		Precision(%)		MPCK20 (%)	Distance (pixels)
PAINet	FL+MSE	$62.69\pm 1.90$	$36.59\pm 5.40$	$84.12\pm 3.70$	$71.12\pm 4.80$	$51.20\pm 2.80$	$138.76\pm 2.30$
HRNetv2	FL+MSE	$63.18\pm 4.59$	$42.65\pm 8.75$	$79.44\pm 2.74$	$69.17\pm 11.22$	$95.35\pm 3.81$	$65.53\pm 10.21$
HRNetv2	CE+MSE	$63.58\pm 4.20$	$\varvec{45.97\pm 5.27}$	$79.67\pm 3.00$	$67.29\pm 7.87$	$97.31\pm 3.70$	$64.13\pm 7.60$
PitSurgRT	0.9Dice+0.1BDL	$\varvec{67.00\pm 4.18}$	$45.92\pm 5.21$	$\varvec{81.3\pm 2.70}$	$\varvec{71.72\pm 8.81}$	$\varvec{97.90\pm 4.01}$	$\varvec{54.48\pm 7.45}$
	+0.8Wing+0.2FL*						

*Denotes resampled. Bold values indicate the best-performing model for that column’s metric

**Table 2:** Ablation studies (single-fold)

Loss	Sella	Clival recess	Sella	Clival recess	Landmarks	
	IoU(%)		Precision(%)		MPCK20 (%)	Distance (pixels)
CE+MSE+BCE	68.01	47.14	73.23	63.20	75.39	102.09
CE+MSE+FL	68.06	49.07	72.98	66.84	86.43	84.05
CE+Wing+FL	66.83	49.34	71.86	65.33	99.22	60.22
CE+Wing+FL*	68.07	49.65	71.99	63.26	99.81	57.85
1.2CE+0.8ABL+Wing+FL*	68.12	49.65	73.83	67.50	99.61	62.94
CE+0.01BDL+Wing+FL*	67.87	47.50	72.42	65.55	99.22	60.00
gDice+0.01BDL+Wing+FL*	66.09	**51**.**29**	80.14	72.39	**100**.**00**	61.43
Dice+0.1BDL+Wing+FL*	69.28	49.06	79.65	68.14	99.81	57.68
0.9Dice+0.1BDL+0.8Wing+0.2FL*	**72**.**06**	47.96	**82**.**51**	**81**.**16**	**100**.**00**	**50**.**15**

*Denotes resampled. gDice stands for generalized Dice. Bold values indicate the best-performing model for that column’s metric. The bottom row is the proposed model, PitSurgRT

**Table 3:** Comparison of model acceleration (fivefold cross-validation, results are displayed as $\mathrm{mean\pm std}$)

Model	IoU(%)		MPCK20 (%)	mDistance (pixels)	Inference time (milliseconds)	Frames per second (fps)
	Sella	Clival recess				
PyTorch model	$67.00\pm 4.18$	$45.92\pm 5.21$	$97.90\pm 4.01$	$54.48\pm 7.45$	$90.18\pm 5.15$	$11.12\pm 0.60$
TensorRT engine (FP32)	${67.00\pm 4.18}$	$45.92\pm 5.21$	${97.90\pm 4.01}$	$54.48\pm 7.45$	$44.13\pm 7.95$	$233.77\pm 39.81$
TensorRT engine (FP16)	${67.00\pm 4.18}$	$45.93\pm 5.23$	${97.90\pm 4.01}$	$54.48\pm 7.45$	$\varvec{33.55\pm 1.65}$	$\varvec{298.81\pm 14.80}$

Bold values indicate the most efficient model for that column’s metric

**Fig. 4:**
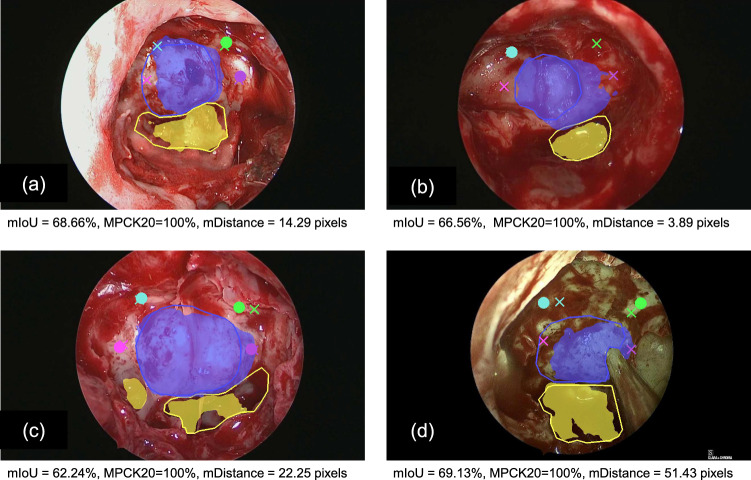
Qualitative evaluation of the predicted results

From Table 1, it is clear HRNetv2 has significantly improved performance over the current state-of-the-art, PAINet. When using the same loss (FL + MSE), there is a small improvement in segmentation performance (+3.28% mIoU) but a significant improvement in landmark detection (+44.15% MPCK20). This is further improved with the proposed weighted loss, with +3.54% segmentation mIoU and +2.55% landmark MPCK20. Although calibration parameters of endoscopes are not available, according to [23], the diameter of the sella is about 10–15 mm, which is around 200–300 pixels in our dataset. Thus, we estimate the scale of our images is about 0.05 mm/pixel. Therefore, according to Table 1, it can be estimated that the accuracy of landmark detection using our method is around 2.35$-$3.10 mm. The ablation studies displayed in Table 2 show the proposed weighted loss is most effective, with a $-$7.53 mDistance pixel improvement over the next best loss. While IoU of clival recess is comparatively low, this is mainly because of this structure (a) not having a well-defined boundary and (b) missing annotations. However, as mentioned in section “Clinical motivation and validation”, clival recess is not considered critically important by the surgeons.

Figure 4 shows the qualitative results on images from four different surgeries also showcasing the inter-patient variability in such procedure. Note that all predictions appeared in anatomically correct regions. Figure 4a and b shows cases where the landmarks align 100% with the groundtruth, and this is also confirmed by the strong agreement among the consultant and trainee neurosurgeons mostly rating these cases as precisely accurate. In Fig. 4c, left optic protuberance did not accurately align with the groundtruth. Figure 4d is the most challenging case, where the predicted landmarks, though within MPCK20, were not 100% aligned with the groundtruth. There were disagreement in surgeons’ rating for this case too, considering the visually different and clinically challenging nature of this case.

While half-precision generally leads to lower performance, it is found that our TensorRT-FP16 model has an over 25$\times$ speed improvement over PyTorch, with no apparent decline in segmentation and detection performance, as displayed in Table 3. The slight improvement of the model with FP16 in the segmentation of the clival recess was due to random factors. The results demonstrate real-time performance of our model is feasible in practice.

**Fig. 5:**
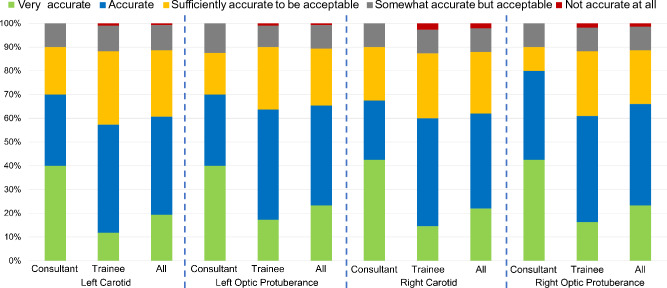
Clinical validation results in which 4 consultant neurosurgeons and 11 trainee neurosurgeons participated to validate the accuracy of PitSurgRT on surgical images from 10 patients

The results from the PitSurgRT output clinical validation study are displayed in Fig. 5. On mean-average, 88.67% of all neurosurgeons agreed the landmark detection outputs were acceptable, increasing to 89.4% of consultants. Less than 1.34% of neurosurgeons (0.00% of consultants) believed the outputs were (1) Not accurate at all. This is because images challenging for PitSurgRT are even difficult for consultant neurosurgeons to identify landmarks on. This is the primary reason ground-truth landmarks were not available for such cases. The clinical validation study demonstrates the success of the network, and that clinical translation is desirable. Moreover, with PitSurgRT already having real-time implementation, feasibility trials in mock operating room settings are planned next. Example images where landmarks were considered accurate and inaccurate to identify are displayed in Fig. 4a and d, respectively.

## Conclusion

The intraoperative localization of crucial anatomical structures in the sellar phase of endoscopic pituitary surgery poses a formidable challenge due to the limited visibility of critical structures, the demand for high accuracy, and the imperative for real-time inference. In this paper, a multi-task model PitSurgRT is proposed to achieve real-time and simultaneous inference of anatomy semantic segmentation and landmark detection with improved accuracy. Compared to the current state-of-the-art, PAINet, PitSurgRT achieved 67.00% (+4.31%) and 45.92% (+9.33%) IoU for sella and clival recess segmentation, respectively. More impressively, it achieved 97.90% (+46.7%) MPCK20 and 54.48 ($-$84.28) mDistance for 4-structure landmark detection. In the clinical validation study comprising 15 neurosurgeons, PitSurgRT demonstrated accurate enough for real-time guidance in 88.67% of cases.

An international Delphi consensus panel determined the phases for endoscopic transsphenoidal pituitary adenoma resection surgery [5]. This process identified that landmark detection is key to the success of this surgery. In this paper, we have presented technological progress in identifying these landmarks. Future work will deploy this system and assess clinical impact.

## Footnotes

1 https://github.com/onnx/onnx.

2 https://developer.nvidia.com/tensorrt.

3 https://github.com/NVIDIA-AI-IOT/torch2trt.
